# Characterization and utility of immobilized metal affinity-functionalized cellulose membranes for point-of-care malaria diagnostics

**DOI:** 10.1016/j.jchromb.2021.123023

**Published:** 2021-12-01

**Authors:** Carson P. Moore, Kristina Pieterson, Jenna M. DeSousa, Lauren E. Toote, David W. Wright

**Affiliations:** a Vanderbilt University, Department of Chemistry, 1234 Stevenson Center Lane, Nashville, TN 37212, USA; b Elizabethtown College, Department of Chemistry and Biochemistry, 1 Alpha Drive, Elizabethtown, PA 17022, USA

**Keywords:** Malaria diagnostics, Immobilized metal affinity chromatography, Cellulose membranes, Sample preparation, Biomarker detection

## Abstract

Immobilized metal affinity chromatography (IMAC) is a well-established technique for protein separation and purification. IMAC has been previously utilized to capture the malaria biomarker histidine-rich protein 2 (HRP2) from blood, enhancing the sensitivity of field-appropriate diagnostic tools such as lateral flow assays. However, little work has been done to translate this technique to a truly field-usable design. In this study, IMAC-functionalized cellulose membranes are created and characterized fully for future use in applied malaria diagnostics. IMAC-functionalized cellulose membranes were investigated across a range of cellulose substrates, IMAC ligands, and divalent transition metals before use in a capture and elution flowthrough workflow. Following characterization and optimization, it was found that iminodiacetic acid bound to Zn(II) was the most promising ligand–metal pair, with three available coordination sites and a molar loading capacity of 57.7 μmol of metal/cm^3^ of cellulose. Using these parameters, more than 99% of HRP2 was captured from a large-volume lysed blood sample in a simple flow-through assay and 89% of the captured protein was eluted from the membrane using the chelating compound ethylenediaminetetraacetic acid. Use of this enhancement protocol on an in-house HRP2 lateral flow assay (LFA) yielded a limit of detection of 7 parasites/μL, a 15.8x enhancement factor compared to traditional LFA methods.

## Highlights

• Malaria diagnostics exist at a unique nexus where robustness and usability intersect with a significant need for increased sensitivity.
• Immobilized metal affinity-functionalized cellulose membranes are a promising tool for increasing the sensitivity of point-of-care malaria diagnostics.
• For the primary malaria biomarker, histidine-rich protein 2, IMAC membranes were found to increase sensitivity by 15.8-fold compared to traditional methods.
• IMAC membranes could also be used for capture and recovery of non-histidine rich biomarkers such as *Plasmodium* lactate dehydrogenase.

## 1 Introduction

Immobilized metal affinity chromatography (IMAC) continues to be a highly-utilized protein separation technique since its inception in the mid-1970′s [1], [2], [3], [4]. IMAC uses metal chelating ligands bound to a solid support such as cellulose, agarose, or silica [5], [6]. These ligands behave as Lewis bases by binding divalent transition metal ions such as copper(II), zinc(II), cobalt(II), nickel(II) or iron(II) [5], [7]. These Lewis acidic metal centers have a natural affinity for accessible, electron-donating amino acids such as histidine, tryptophan, or cysteine allowing for the reversible isolation of peptides or proteins containing these residues from more complex solutions. These residues are often referred to as Porath’s triangle, in recognition of Porath’s pioneering work utilizing these groups in IMAC research [7], [8]. Of the common residues in Porath’s triangle, exposed histidine moieties have been shown to have the highest affinity for the metal ions, and IMAC has become a standard method for the separation or purification of His-tagged proteins [9]. Once isolated on the solid support, proteins can then be eluted from the metal complexes by several methods, including competition with a high affinity molecule such as imidazole or total chelation of the metal ion by a stronger chelating compound such as ethylenediaminetetraacetic acid (EDTA) [10].

Selectivity can vary greatly depending on the combination of IMAC ligand, metal ion, and solid support utilized [11], [12], [13], [14]. Chelating ligands with different denticities, such as tridentate iminodiacetic acid (IDA) or tetradentate nitrilotriacetic acid (NTA), yield different available binding sites [8]. Traditionally, tridentate ligands coordinate to the transition metal of interest with three coordination sites, leaving three sites available on the metal for coordination to the histidine residues of the peptide or protein. Alternately, tetradentate ligands bind through four initial coordination sites to the metal, leaving two sites free to coordinate to histidine residues. The different binding site availability between ligands can affect both metal binding and downstream protein retention. Metal ion choice can also significantly affect the ability of IMAC systems to bind peptides or proteins of interest. Certain “soft” metal ions, such as Cu(I), Cd(II), or Pb(II), display preferential binding to sulfur atoms, whereas “hard” Lewis acids, including group II and III elements, preferentially chelate oxygen atoms [7]. Borderline transition metals, which are the most common IMAC metals, have combined hard and soft characteristics, and can coordinate to nitrogen, oxygen, and sulfur atoms interchangeably. Among these metals, there are varied levels of coordination capability, and the selection of a specific metal can drastically affect protein retention and recovery.

These separation techniques lend themselves to the diagnosis of malaria, through binding of histidine-rich protein 2 (HRP2), the primary biomarker of *Plasmodium falciparum* malaria [15]. Most commercially-available malaria rapid diagnostic tests (RDTs) detect HRP2 by capturing the protein at a test line using HRP2-specific antibodies. Once the protein is immobilized at the test line, another orthogonal HRP2-specific antibody, bound to a gold nanoparticle, binds to the protein and creates a visual signal [5], [16], [17]. As HRP2 consists of 34% histidine residues, primarily in AHH and AHHAAD sequence repeats, it is an ideal target for an IMAC-based enhancement strategy [18], [19]. Additionally, HRP2 is a versatile biomarker, and has been utilized both as a standalone *P. falciparum* biomarker or in tandem with pan-malaria biomarkers such as *Plasmodium* lactate dehydrogenase (*P*LDH).

However, there is significant variation in sensitivity of RDTs that rely on these biomarkers at low parasite burdens. The World Health Organization has recommended that commercially available RDTs be able to detect parasite burdens of at least 200 parasites per μL of whole blood [20]. While this limit of detection is appropriate for most high-burden infections, recent models have shown that a diagnostic threshold of 200 parasites per μL is only sufficient for detecting 55% of the total infectious reservoir [21]. Increased sensitivity, for instance by 10-fold or 100-fold, would increase the percentage of the infectious reservoir being detected by 28% or 40% respectively. Thus, investigators have begun to seek additional sample preparation methods to enrich biomarkers of interest to within this range.

The selectivity of different metals on commercially-available resin beads [13] and magnetic beads [14] have been investigated in the context of point-of-care sample preparation method for improved malaria diagnosis. These studies use the innate histidine-rich composition of HRP2 and artificially histidine-enriched antibodies to concentrate malaria biomarkers from large volume blood samples for detection on lateral flow assays (LFAs), the most commonly used malaria RDT. A simpler, inexpensive, more integrated approach is needed to reach areas that remain completely underserved by any traditional healthcare or laboratory infrastructure. In this study, we have developed and fully characterized a paper membrane-based IMAC system that has been designed to isolate and enrich the histidine-rich malaria biomarker HRP2 from lysed whole blood. This system represents a critical advancement in simple-to-use, field-appropriate separation science.

## 2 Experimental

### 2.1 Materials

Cellulose membranes (Whatman chromatography paper Grades 1 Chr, 2 Chr, 3 Chr, 4 Chr, 17 Chr, 3MM, and Whatman Grade 3 filter paper), nitrocellulose membranes (FF80HP, FF120HP), and wicking pads? (CF7) were purchased from Cytiva Life Sciences (Marlborough, MA, USA). The flow properties of the Whatman cellulose membranes are described in Table 1. 40 nm unconjugated gold colloids were purchased from Ted Pella Inc (Redding, CA, USA). Anti-HRP2 antibodies, ABMAL 0405 IgM and ABMAL 0404 IgG, were purchased from Arista Biologicals Inc (Allentown, PA, USA). The anti-*P*LDH IgM capture antibodies used (clone 19 g7) were purchased from Vista Laboratory Services (Langley, WA, USA). Control goat-anti-mouse antibodies were purchased from Fitzgerald Industries International (Acton, MA, USA). Pooled gender human whole blood in citrate phosphate dextrose (CPD) anticoagulant was purchased from BioIVT (Westbury, NY, USA). All other reagents were reagent grade, purchased from Fisher Scientific and Sigma Aldrich.

**Table 1:** Manufacturer-reported flow properties for all screened Whatman cellulose membranes. (NR = not reported).

*Membrane*	*Membrane Type*	*Thickness (μm)*	*Reported Linear Flow Rate (mm/30* min*)*	*Reported Gurley Air Permeability (s/100 mL/in^2^)*
1 Chr	Chromatography	180	130	11
2 Chr	Chromatography	180	115	20
3 Chr	Chromatography	360	130	26
4 Chr	Chromatography	210	180	3.67
3MM	Chromatography	340	130	20
17 Chr	Chromatography	920	190	9
Grade 3 FP	Filter paper	390	*NR*	26

### 2.2 Cellulose membrane functionalization

The cellulose membrane functionalization method was adapted from Ke et al. [22] Briefly, a 5 cm by 5 cm square of cellulose was submerged in a 25 mL solution of 80% 1.6 M sodium hydroxide, and 20% epichlorohydrin. The membrane was incubated in solution for 8 h, before washing 3 times with ultrapure water over vacuum filtration. The washed membrane was then incubated overnight in 25 mL of 1 M chelating ligand (iminodiacetic acid (IDA) or 2,2′-((5-amino-1-carboxypentyl) azanediyl) diacetic acid (NTA-lysine)) and 1 M sodium carbonate. The NTA-lysine derivative was synthesized in-house (Supplemental Methods 1.1), and used in place of pure commercial NTA in order to maximize the binding capacity of metal ions. The addition of a lysine arm to the NTA molecule prevents membrane epoxide groups binding through an NTA dentate arm during membrane functionalization. Following the overnight incubation, the membrane was again washed 3 times with ultrapure water. The membrane was then incubated in a 25 mM solution of metal sulfate for 1 h before washing 3 times and drying at room temperature. All membranes were stored at room temperature. Before use in flowthrough experiments, functionalized membranes were cut to 12 mm by 12 mm squares.

### 2.3 Membrane flow time characterization

Functionalized membrane vertical flow time was investigated by pipetting 500 μL of lysed blood onto a dry 12 mm by 12 mm square of membrane. Binder clips were used to hold the membrane in a sample well, and the time from addition of blood to complete flow through the membrane was measured, as determined by the sample emergence in the sample well and the drying of the visible surface of the membrane. This measurement was then repeated for a mock elution sample. The mock elution samples were made by flowing a 500 μL sample of 100 mM EDTA through the membrane following the addition of the lysed blood. The time from addition to complete flowthrough was measured as before.

### 2.4 IMAC metal loading quantification

The amount of metal bound to each membrane was quantified using an Optima 7000 DV inductively coupled plasma optical emission spectrometer (ICP-OES). Samples of membrane were collected using a 6 mm biopsy punch. Each 6 mm circle of membrane was then digested in 14% TraceMetal™ grade nitric acid for 15 min. The cellulose and nitric acid solution was then filtered through a 0.45 μm PVDF filter, and diluted to 2% nitric acid. Samples were then analyzed against a series of known standards and measured at 228.616 nm (Co(II)), 206.200 nm (Zn(II)), 231.604 nm (Ni(II)) and 224.700 nm (Cu(II)).

### 2.5 Scanning electron microscopy (SEM) binding analysis

The binding capabilities of the metal ligands were investigated using energy dispersive spectroscopy (EDS) on a Zeiss Merlin scanning electron microscope (SEM). Membranes were synthesized as described previously, but were washed with a solution of 25 mM L-cysteine before drying. A 12 mm by 12 mm square of cysteine-washed membrane was affixed to a 12.7 mm diameter sample mount. All SEM samples were stored in desiccators after synthesis to maximize compatibility with the Zeiss SEM. Before analysis, all membranes were sputtered with gold for 80 s at a stage height of 57.5 mm. SEM/EDS analysis was performed at various magnifications using a 10 kV beam voltage for IDA samples and 20 kV for NTA-lysine samples.

### 2.6 HRP2 protein quantification

To prepare the samples, two methods were utilized. In the initial sample preparation method, a 1 cm diameter circle of functionalized membrane was placed in the bottom of a 2 mL plastic syringe. Human whole blood was spiked with D6 parasite culture (stock parasitemia: 43,600 parasites/μL) and lysed 1:1 with lysis buffer (phosphate buffer, pH = 8, 300 mM NaCl, 2% Triton 100). 500 μL of parasitized lysed blood with a final parasitemia of 25p/μL was added to the membrane within the syringe and allowed to flow vertically for one minute before being flushed with the plunger. The resulting supernatant was collected in an Eppendorf tube. Next, a 500 μL aliquot of 100 mM EDTA was added to the membrane within the syringe and similarly allowed to flow by gravity filtration through the membrane for one minute before being flushed through with the plunger. The eluent was collected in a separate Eppendorf tube. All experiments were performed in method triplicate, and both the capture supernatant and eluent were quantified by the *Pf*HRP2 ELISA developed by Davis *et al*. [23].

To more realistically mirror a potential field-based RDT setting, a second HRP2 capture workflow was developed. In this method, a 12 mm by 12 mm square of IMAC-functionalized membrane was placed into a membrane holder and secured with binder clips. A 500 μL sample of parasitized lysed blood was pipetted onto the membrane and allowed to completely flow through the paper into the sample collection well. The sample was removed from the well before 500 μL of elution buffer (100 mM ethylenediaminetetraacetic acid, EDTA) was pipetted onto the paper and allowed to flow through into the sample well. All collected samples were analyzed using the *Pf*HRP2 enzyme-linked immunoassay (ELISA) developed by Davis et al. [23] Capture and elution values were evaluated for statistical significance using a one-way ANOVA test.

### 2.7 On-membrane capture and elution of PLDH

Anti-PLDH antibodies were prepared using the protocol provided by Bauer et. al. [13] Briefly, 19 g7 anti-*P*LDH IgM antibodies were activated with sulfosuccinimidyl 4-(N-maleimidomethyl) using the Sulfo-SMCC No Weigh Format aliquots (Thermo Scientific, A39268) at a 20x excess. Excess Sulfo-SMCC was removed using a 7 k molecular weight cutoff (MWCO) desalting column before addition of an in-house His_6_-biotin peptide. The peptide was also added at 20x molar excess. After incubation with the peptide, excess His_6_-biotin was removed using the 7 k MWCO desalting columns and the histidine-enriched antibodies (hAb) were stored at −20 °C.

To determine capture of *P*LDH from lysed blood, increasing concentrations of hAb were added to a 12x12 mm square of Zn(II)-IDA functionalized 17 Chr membrane, and allowed to flow through completely. 500 μL of parasitized lysed blood was then added to each membrane, and the supernatant was collected to quantify *P*LDH not captured by the membrane. *P*LDH quantification was performed using the ELISA described by Bauer et al. [13] Eluted *P*LDH was quantified using 500 μL of 100 mM EDTA, and supernatant was collected for analysis by the *P*LDH-specific Malstat assay described by Markwalter et al. [24].

### 2.8 Lateral flow assay development

The HRP2 LFA was fabricated using a BioDot CE 1520 Array and Dispense Platform (BioDot, Irvine, CA). Anti-HRP2 IgM antibodies were immobilized at a concentration of 1 mg/mL in a horizontal stripe across a fast-wicking polystyrene-backed nitrocellulose membrane. Goat-anti-mouse IgG antibodies were then deposited in a parallel line downstream of the HRP2 test line. Tests were allowed to dry for 2 h at 37 °C before being blocked with 5 mL of Pierce Protein Free blocking buffer. After blocking, test strips were dried for 3 h at 37 °C, and then cut into 5 mm strips using a BioDot CM 4000 high-precision lateral flow assay cutter. All tests were stored in a desiccated environment at room temperature before final assembly with wicking pad.

LFA conjugate was produced by incubating unconjugated OD1 40 nm gold colloids with ABMAL 0404 anti-HRP2 IgG in 50 mM borate buffer for 1 h on an orbital shaker. The gold colloids were then blocked with 10% bovine serum albumin (BSA, w/v) in 50 mM borate buffer for 1 h before centrifugation at 2500 g at 4 °C for 30 min. After centrifugation, the supernatant was removed, and the pellet was washed with a diluent buffer of 1% BSA and 10% Trehalose (w/v) in 50 mM borate buffer. The solution was then centrifuged again under the same conditions, the supernatant was removed, and the pellet was diluted to a final OD of 10.

### 2.9 Assessment of integrated IMAC LFA

The LFAs were analyzed by comparing the enhanced IMAC-integrated LFA to a traditional LFA. Enhanced LFAs were performed using a titration series of parasite culture spiked into lysed whole blood and the sample preparation method detailed above. 10 μL of OD 10 anti-HRP2 conjugate was added to 125 μL of the mock elution sample, then an in-house LFA was dipped into the mixture and allowed to wick upwards for 15 min. The LFAs were then transferred to a running buffer of 50 mM borate buffer + 0.1% Tween 20 for 3 min to assist in membrane clearing.

Traditional LFAs were run as vertical dipstick tests, and consisted of a 10 μL sample of parasite-spiked lysed whole blood added to 120 μL of running buffer (1x borate buffer with 0.1% Tween 20)**.** 10 μL of conjugate was added to the sample and an LFA was added to the mixture and allowed to wick for 15 min, followed by a 3-minute wash step.

Both IMAC-enhanced and traditional lateral flow test strips were analyzed on a Qiagen ESEQuant lateral flow reader (QIAGEN Lake Constance GmbH, Germany). The average test line peak area and standard deviation were calculated at each parasite density in the titration series and the limit of detection was calculated using 3SD_blank_ divided by the slope of the regression of the linear region of the data.

## 3 Results and discussion

**Fig. 1:**
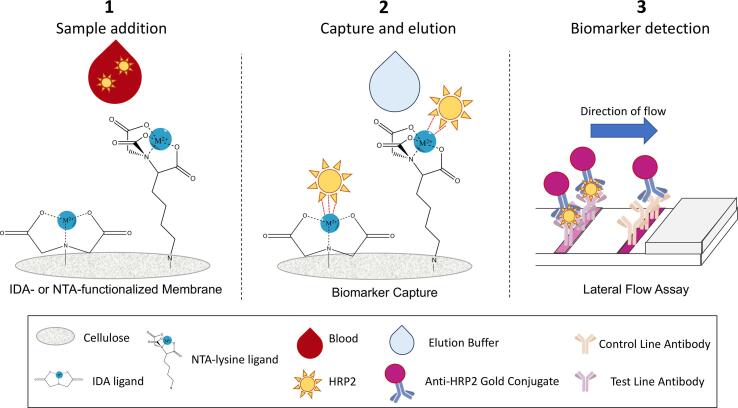
Workflow of IMAC-cellulose sample preparation: (1) Cellulose membranes are functionalized with either IDA or NTA metal affinity ligands. (2) Metal affinity ligands capture HRP2 from a large-volume sample (3) The HRP2 eluted from the membrane can then be detected colorimetrically using an immunoassay such as a lateral flow assay (LFA, shown above) or enzyme-linked immunosorbent assay (ELISA).

In this manuscript, we are presenting a method for enriching malaria biomarker using an easily-produced, inexpensive cellulose membrane that could be incorporated into devices directly at the point of care (Fig. 1). This membrane is innately capable of utilizing IMAC binding to capture histidine-rich malaria biomarkers. The membrane can also be used to immobilize non-histidine-rich biomarkers such as *P*LDH through the use of artificially histidine-enriched antibodies. However, for this proof-of-concept study, we have chosen to focus on the capture and elution of histidine-rich biomarkers.

### 3.1 IMAC-Functionalized cellulose characterization

The binding capacity for both IDA and NTA was investigated using SEM/EDS to measure the ratio of L-cysteine, represented by the single detectable sulfur atom of the cysteine moiety, to the divalent metal on a Whatman Grade 3 FP membrane. In theory, tridentate ligands such as IDA use three atoms for metal chelation, leaving three available binding sites on the metal center for binding protein in solution. Tetradentate ligands such as NTA occupy four of the six binding sites in the coordination sphere, which leaves two available for protein binding. It was observed that, in agreement with the theoretical value, IDA-functionalized membranes were capable of binding L-cysteine to the membrane at a 3:1 ratio of sulfur to divalent metal (Table 2). However, it was also observed that the NTA-functionalized membranes bound at 2.72:1 ratio, slightly higher than the expected value of 2:1. This is likely due to steric inhibition of the coordination sites on the NTA-lysine molecule. The NTA-lysine derivative was selected in place of standard NTA to prevent binding of the on-membrane epoxide through one of the dentate arms, thus preserving the four distinct coordination sites. However, the addition of the lysine tail does not remove the other potential reaction sites within the NTA-lysine molecule. Therefore, it is possible that the ligand bound to the membrane in such a way that appropriate binding of the divalent metals was prevented by epoxide residues binding the NTA-core, rather than reacting through the nitrogen group of the tail-end lysine as expected. Thus, the average ratio of sulfur atoms to M(II) could be slightly higher than expected. This steric hinderance and ligand misalignment is also likely responsible for the lower concentration of metal observed on NTA-lysine-functionalized membranes compared to IDA-functionalized membranes, as metal ions would be impacted by the misaligned binding of the NTA-lysine ligand.

**Table 2:** Observed SEM-EDS weight percent values for the primary elements in IDA- and NTA-functionalized membranes washed with L-cysteine, where M (II) refers to the divalent metal.

	**Weight Percent (%)**	
**Element**	*IDA*	*NTA*
C	49.5 ± 0.1	52.95 ± 0.05
O	43.9 ± 0.1	46.30 ± 0.05
S	4.8 ± 0.1	1.17 ± 0.01
M(II)	1.6 ± 0.1	0.43 ± 0.01
S:M(II) ratio	*3:1*	*2.72:1*

**Fig. 2:**
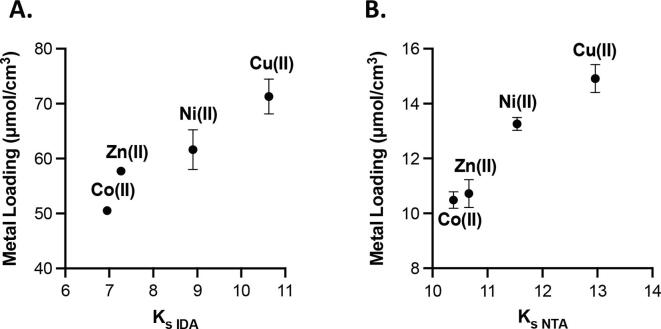
Metal loading on 17 Chr cellulose with each divalent metal ion using the IDA ligand (A) and NTA ligand (B) compared to the reported stability constant K_s_ for each divalent metal ion. Some error bars are not shown as the error lies within the point.

Next, metal loading capacity, which here is defined as the molar quantity of M(II) bound to the cellulose membranes, was probed for each combination of IDA or NTA and the 4 most common divalent IMAC metals: cobalt (II), nickel (II), copper (II), and zinc (II) using ICP-OES. The ICP-OES experiments were performed using Whatman 17 Chr chromatography paper as the cellulose substrate, as it is easily available and amenable to use in flow-based devices based on the reported flow rate and air permeability (Table 1). As expected, all metals were found to bind successfully to the cellulose substrate, further, IDA-functionalized ligands bound significantly more metal per cubic centimeter of membrane than NTA-lysine-membranes, (Fig. 2A and 2B). When the quantity of metal loaded on the membrane was plotted against the theoretical stability constant, K_s_, for each metal–ligand pair, there was a clear trend of increasing loading capacity with increasing K_s_. This trend validates the expected affinity between each of the transition metals and the respective chelating ligand, as the highest reported K_s_ is directly correlated to the highest observed metal loading capacity for each ligand.

**Fig. 3:**
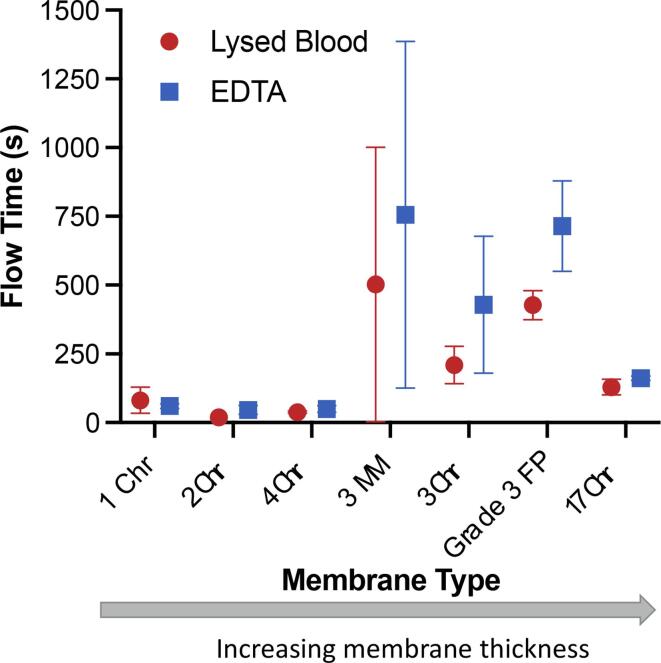
Time elapsed to complete vertical flow of a 500 μL sample of lysed blood and 500 μL 100 mM EDTA using different cellulose membranes of increasing thickness.

Each of the 7 potential membrane types were then examined with respect to their ability to process a sample via vertical flow (Fig. 3), in preparation for use in a vertical protein capture and elution scheme (Fig. 1). Thin membranes such as 1 Chr, 2 Chr, and 4 Chr chromatography papers (160 μm, 180 μm, and 210 μm thick respectively) retained very little liquid sample with minor variations in flow, resulting in consistent vertical flow through the membrane in a matter of seconds. Membranes of mid-range thickness, such as 3MM chromatography paper (340 μm), 3 Chr chromatography paper (360 μm), and Grade 3 FP (390 μm) had significantly longer flow times than the thinner membranes and showed significant variation, with maximum flow times exceeding 4 h (these points were considered outliers based on a Q-test for n = 3 at 90% CI and omitted from analysis). Additionally, these middle-grade thickness membranes, which also had similar porosity values, appeared prone to clogging from blood residue and cell debris, causing significant increases in elution flow rate compared to thinner membranes. Interestingly, the thickest membrane, Whatman 17 Chr (920 μm) demonstrated remarkably fast vertical wicking speed for both blood and elution buffer and had little variation compared to the middle-thickness membranes. This is consistent with its reported linear flow rate (190 mm/30 min) and Gurley permeability (9 sec/100 mL/in^2^) (Table 1).

### 3.2 On-Membrane HRP2 capture and recovery

HRP2 capture and recovery were assessed using a previously established ELISA protocol for HRP2-specific detection in human blood samples, as opposed to other methods that could be used to quantify biomarker enrichment such as SDS-PAGE [23]. This particular method has been validated in low-resource field settings, and was found to have a specificity of 89% when compared to microscopy, the current gold standard for laboratory diagnosis of malaria in the field [25]. Additionally, as ELISAs rely on the detection of proteins of interest through the formation of immunocomplexes with antigen-specific antibodies, we believe that this technique is sufficient to specifically detect HRP2 with little to no interference from nonspecific blood proteins.

**Fig. 4:**
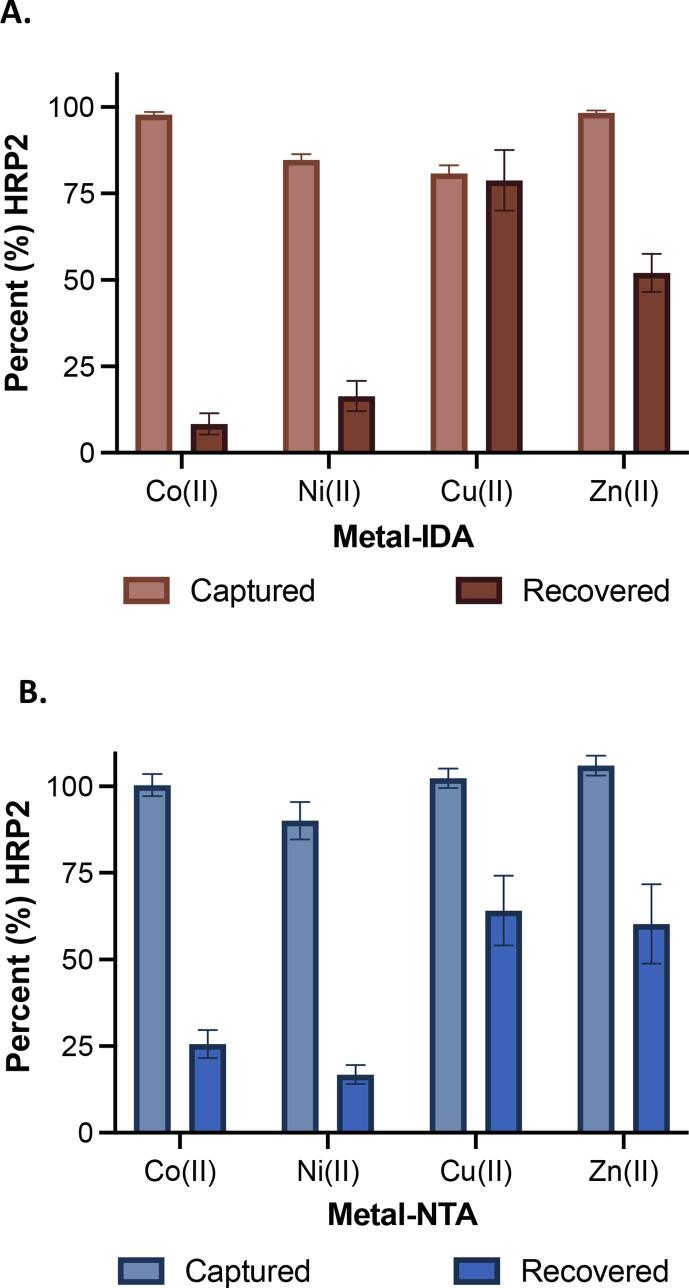
Percent HRP2 captured and recovered using each divalent metal ion bound to either the IDA ligand (A) and NTA ligand (B).

First, the ELISA was used to confirm that the entire ligand–metal system, as opposed to ligand or epoxide alone, was necessary for HRP2 binding (Supplemental Fig. 1). Then, each of the ligand–metal combinations were tested for HRP2 binding capacity in a membrane-based vertical flowthrough assay using the 17 Chr cellulose membrane as a starting point. While all metal complexes yielded high HRP2 capture percentages (greater than80%), the recovery values varied widely (Fig. 4). For IDA membranes, the capture among the four transition metal ions was statistically different (p < 0.0001, 95% CI), with Cu(II) capturing the lowest percentage of HRP2 and Zn(II) and Co(II) capturing more than 99% of the protein in solution. Using NTA-lysine, Co(II), Cu(II) and Zn(II) are not statistically distinct (p = 0.1403, 95% CI) while Ni(II) has a distinctly lower capture percentage (Fig. 4B). For both IDA and NTA ligands, Cu(II) and Zn(II) were shown to have the highest protein recovery. Using Cu-IDA membranes, 78.8% ± 8.8% of the total protein was recovered, whereas 52.0% ± 5.5% of the total HRP2 was recovered using Zn-IDA preparation. For the NTA-lysine-functionalized membranes, protein recovery was not improved for Cu(II) or Zn(II) (64.1% ± 10.1% and 60.2% ± 11.5%, respectively). Although there is not a large difference in capture efficiency among the four metal ions, the high capture efficiency of Co(II) and Zn(II) IDA membranes reflects the k_on_ observed in literature [13]. k_on_, or the second order association rate constant, defines the rate of formation of a given complex over time. Thus, the high k_on_ values reported for both Co(II) and Zn(II) were found to be in accordance with the expected results. However, Cu(II) was previously reported to have the least desirable kinetic parameters when examined in the context of resin-based solution separation and biolayer interferometry, but here shows the most effective protein recovery. Cu(II) was observed to have the lowest capture percentage, consistent with the biolayer interferometry results in the literature, as it has the lowest reported k_on_ value. Cu(II) was also reported to have a high K_D_ value. A small K_D_ value, or equilibration constant, indicates tight binding and high retention of the protein of interest, thus a higher K_D_ value, such as observed with Cu(II), supports the dissolution of the Cu(II)-HRP2 complex and higher proportion of protein recovery observed.

However, Cu(II) was down-selected in this process as Cu(II)-functionalized membranes showed adverse effects when treated with lysed or whole blood (Supplemental Fig. 2). Upon coming into contact with Cu(II)-treated membranes, blood samples underwent a visible shift in color from red to brown and precipitated solid in the sample. Additionally, it was found that the presence of Cu(II) in small concentrations interfered with the results of immunogenic assays (Supplemental Fig. 3). Thus, although Zn(II)-functionalized membranes resulted in slightly less protein recovery, the high capture percentage and lack of bioreactivity of the Zn(II) membranes made these a natural choice for further development.

**Fig. 5:**
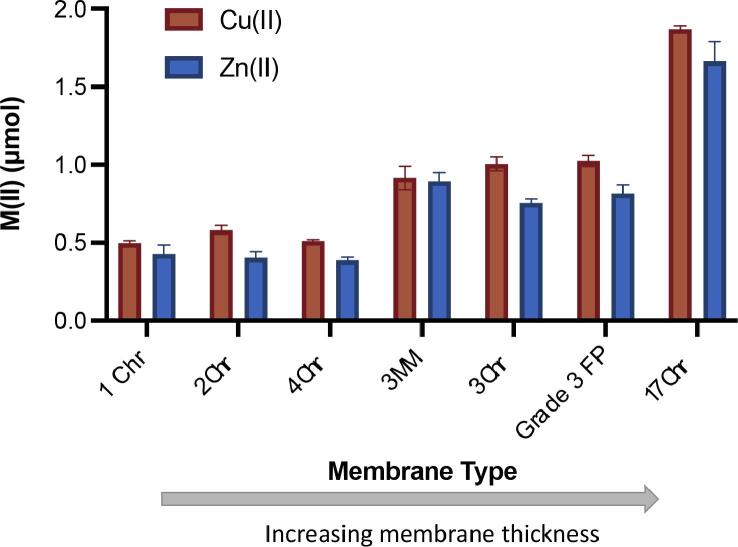
Zinc and copper ion loading for seven different cellulose membranes by increasing membrane thickness.

Metal loading and HRP2 binding were then investigated for Cu(II) and Zn(II) using the 7 distinct cellulose membrane types detailed in section 3.1 (Fig. 5). When compared by membrane thickness or area, there was a distinct increase in metal loading as expected based on the parameters outlined in Table 1.

**Fig. 6:**
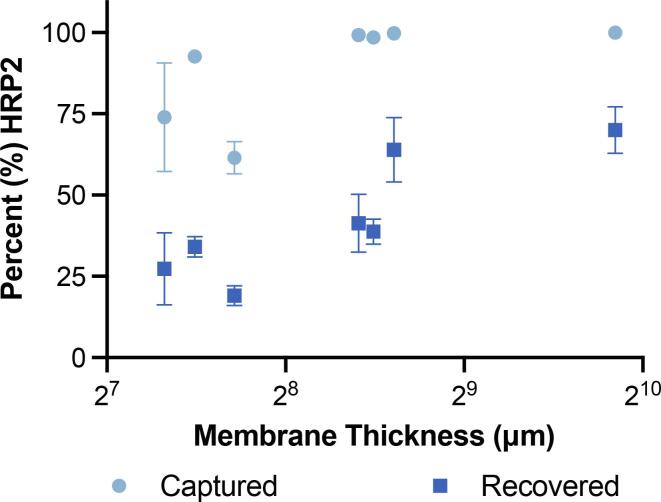
Percent HRP2 captured and recovered using Zn(II)-IDA functionalized membranes of increasing thickness.

Following the selection of Zn(II) as the ideal candidate metal for on-membrane protein enrichment, each of the seven membrane types characterized previously was investigated for HRP2 binding capacity using Zn(II)-IDA functionalization (Fig. 6). IDA was utilized as the preferred IMAC ligand due to the ease of availability compared to NTA-lysine, as well as lower observed variation for capture using Zn(II). 17 Chr, the thickest and fastest-flowing membrane, outperformed the six other membranes tested, thus remaining the ideal candidate for use in LFA enhancement work. Despite Grade 3 FP showing similar recovery percentages for HRP2, it was down selected due to significantly worse practical flow time, making it an inappropriate substrate for a quick, field-usable diagnostic test. Additionally, while all membranes above 300 μm thick were observed to capture 100% of the protein from the sample, 69.9% ± 7.2 of the total protein was recovered after elution using the 17 Chr cellulose membrane. The high proportions of both captured and recovered protein were expected due to the high concentration of M(II) observed on the thick 17 Chr membranes, allowing for more interaction between sample and elution buffer during flow.

Additionally, it is important to note that all capture and elution experiments were performed using the syringe method as a proof-of-concept. This method, which allows a single minute of gravity flow before expulsion from the syringe using the plunger, does not allow the sample to fully interact with the membrane as it would during vertical flow in a field-based setting. Further, the plunging and refilling of the syringe represents a multi-step process that would further limit the utility of this format at the point of care, as well as an additional variable that can affect performance. It was observed during these experiments that the insertion of the membrane into the syringe, as well as the membrane’s displacement during the plunging step before elution, could, on occasion, result in folds in the membrane or the disruption of the seal around the cellulose. Thus, using this format, it was found that the recovery of HRP2 was lower than expected in some cases, as the eluent buffer was able to flow around the membrane as opposed to flowing through the functionalized cellulose. It is expected that in a traditional vertical flow design, which does not have the drawbacks of the current design and relys only on the sample’s vertical flow through the membrane, would have increased capture and elution ability compared to the syringe-based system. Additionally, it may be possible that with increased ambient flow time, as evidenced by the innate vertical flow speeds of the membranes used in this application, the capture and elution efficacy in a purely vertical flow device would be enhanced. Thus, in evaluating the enhancement with regard to LFAs, the membrane holder was used to better mimic an integrated, hands-free, low-effort flow through system.

### 3.3 Lateral flow assay enhancement

**Fig. 7:**
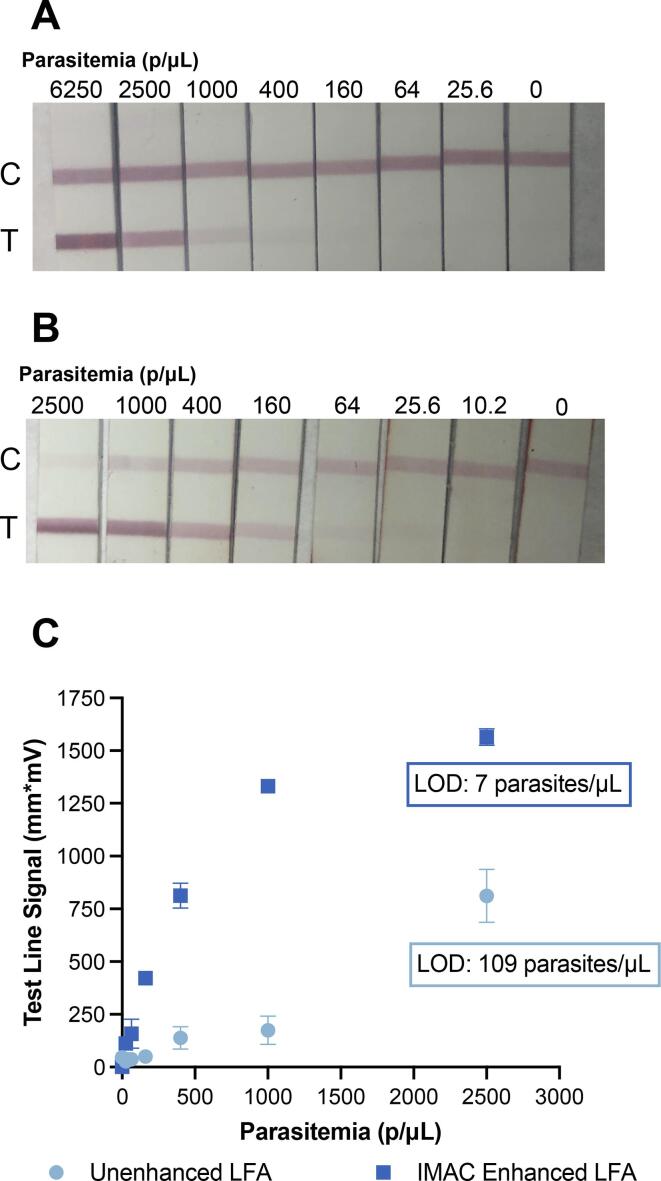
Limit of detection analyses were performed for the unenhanced (A) and IMAC-enhanced (B) LFA preparation using an 8-point spiked parasite titration series diluted by a factor of 2.5. The IMAC-enhanced LFAs were observed to have an improvement factor of 7.5x compared to the traditional LFA method when utilizing test line signal as the metric of interest (C).

The unenhanced LFAs were performed using the highest possible volume of lysed blood as determined in the literature, 10 μL. [26] Using this value, the limit of detection (LOD) of the traditional LFA was found to be 109 parasites/μL (Fig. 7) when using raw test line signal as the metric of interest. This value represents a limit of detection significantly below the WHO accepted value for field-deployable tests of 200 parasites/μL, however it is still not sufficient for detection of asymptomatic disease.

In comparison, the IMAC-enhanced LFAs were observed to have a LOD of 7 parasites per μL when using test line signal as the deciding metric. This results in a 15.8-fold enhancement compared to the unenhanced LFA. For enrichment of a 500 μL sample of lysed blood compared to a traditional 10 μL lysed blood sample, the enrichment factor is 50-fold. However, in this study, a 125 μL aliquot of the eluted HRP2 was used on the 5 mm test strips, as it was found to be the maximum usable sample volume for these test strips. Additionally, the entire 500 μL sample volume was not recovered from the membrane. Due to the absorbent properties of the thick cellulose, there was on average, a remaining volume of 364 μL recovered from the membrane after flowthrough (n = 12) rather than a full 500 μL aliquot. Thus, the 125 μL sample can be considered 34.3% of the total recoverable sample. With this in consideration, the true theoretical enhancement factor for this study is 17.2-fold compared to the unenhanced test, meaning that the IMAC-enhanced enrichment performed with 91.9% efficacy. However, a loss of control line signal is observed in the enhanced diagnostics (Fig. 7B), likely due to the significant enrichment of HRP2. The enriched HRP2, even if not bound at the test line, could bind anti-HRP2 gold conjugate in such a manner that it impedes the binding of goat-anti mouse antibodies at the control line. This is easily remedied by the addition of more gold nanoparticle conjugate, or by using a different control line molecular recognition element. Regardless, the limits of detection observed fall well within the proposed range of asymptomatic malaria parasitemias and could further be improved by using the full enriched sample volume.

**Fig. 8:**
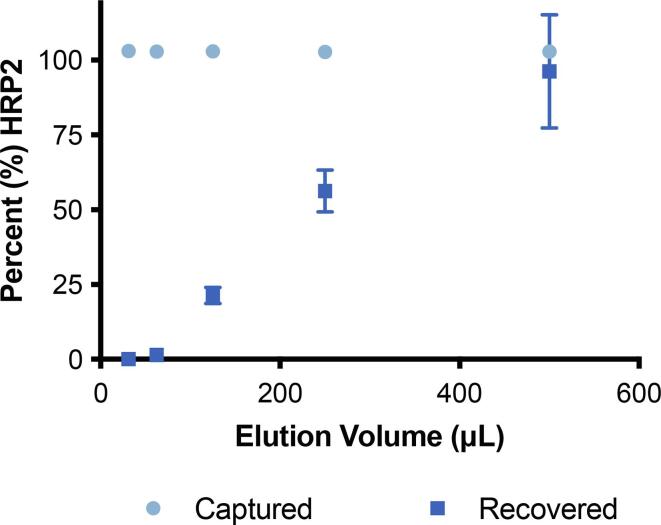
Effect of decreasing elution buffer volume on protein recovery using Zn-IDA-functionalized 17 Chr membranes.

To determine whether a smaller volume of elution buffer could be utilized, thus negating the loss of sample due to the unused portion of the recovered aliquot, a titration series was performed with increasing volumes of EDTA at 100 mM (Fig. 8). It was found that 500 μL of buffer was the optimal volume for protein recovery, and that at lower volumes using the same concentration of EDTA, protein recovery declines significantly. Additionally, as the membrane has been shown to be thick and absorbent, at lower volumes of elution buffer, a significant amount of the buffer was observed to be retained within the membrane.

There are some limitations to this study, including the persisting volume limitations of LFAs. Although we were able to utilize a full 125 μL aliquot of the eluted HRP2 on our 5 mm test strips, there was an average remaining volume of 239 μL recovered from the membrane after flowthrough. Further work should be dedicated to enhancing LFA wicking capacity such that an entire elution sample could be utilized. Additionally, there was a wash step built into the LFA protocol which assisted in clearing blood from the membrane before reading on the LFR. This step is unlikely to be used in a field-based setting, however, a partitioned LFA cartridge could potentially make it possible as a further improvement to the technology available.

### 3.4 Non-histidine rich protein capture and enrichment

Additionally, the membrane was probed for potential use in non-histidine rich protein capture as a proof-of-concept for future development (Supplemental Methods 1.3). Using antibodies against the pan-*Plasmodium* biomarker *Plasmodium* lactate dehydrogenase (*P*LDH), the membrane was shown to have high capture (78.7% ± 11.2) and recovery (57.4% ± 26.1) of the *P*LDH present in a lysed blood sample (Supplemental Fig. 5, Fig. 6). However, limitations of antibody-based LFA development for *P*LDH, outlined in the literature, may hinder this method [13]. Future work examining alternative methods, such as anti-*P*LDH aptamers, may prove beneficial to these applications. [27]

## 4 Conclusions

IMAC resins and beads have been a popular method of protein separation for over 40 years. However, despite their widespread use in laboratory settings, little work has been done to make these techniques appropriate for testing outside of the laboratory, particularly in the realm of biologic sample enrichment for disease diagnosis. In this work, we have optimized a cellulose membrane-based separation technique for the highly histidine-rich malaria biomarker HRP2, and shown potential utility outside of innately histidine-rich biomarkers, as utilized in the enrichment of *P*LDH. We have shown the effectiveness of this technology as a paper-based supplement for large-volume field-based diagnostic tools, a critical need for disease control worldwide. As a large-scale system, this technology could potentially be applied to any number of potential biomarker-based assays for use in field care, enabling biomarker enrichment and improved sensitivity directly at the point of care.

### CRediT authorship contribution statement

**Carson P. Moore:** Conceptualization, Methodology, Validation, Formal analysis, Investigation, Writing – original draft, Visualization. **Kristina Pieterson:** Investigation, Validation, Writing – review & editing. **Jenna M. DeSousa:** Investigation, Resources, Writing – review & editing. **Lauren E. Toote:** Conceptualization, Methodology, Writing – review & editing. **David W. Wright:** Supervision.

## Declaration of Competing Interest

The authors declare that they have no known competing financial interests or personal relationships that could have appeared to influence the work reported in this paper.
